# High Blood Pressure Is Associated with Tubulointerstitial Damage along with Glomerular Damage in Glomerulonephritis. A large Cohort Study

**DOI:** 10.3390/jcm9061656

**Published:** 2020-06-01

**Authors:** Claudio Bazzi, Teresa M Seccia, Pietro Napodano, Cristina Campi, Brasilina Caroccia, Leda Cattarin, Lorenzo A Calò

**Affiliations:** 1 D’Amico Foundation for Renal Disease Research, 20145 Milan, Italy; claudio.bazzi@alice.it; 2 Hypertension Unit, Department of Medicine—DIMED, University of Padova, 35128 Padova, Italy; teresamaria.seccia@unipd.it (T.M.S.); brasilina.caroccia@unipd.it (B.C.); 3 Nephrology and Dialysis Unit, Azienda Ospedaliera Ospedale San Carlo Borromeo, 20153 Milano, Italy; napodano.pietro@sancarlo.mi.it; 4 Department of Mathematics, University of Padova, 35121 Padova, Italy; cristina.campi@unipd.it; 5 Nephrology, Dialysis and Trasplantation Unit, Department of Medicine—DIMED, University of Padova, 35128 Padova, Italy; leda.cattarin@gmail.com

**Keywords:** glomerular, tubulointerstitial damage, kidney, hypertension, proteinuria

## Abstract

The key role of arterial hypertension in chonic kidney disease (CKD) progression is widely recognized, but its contribution to tubulointerstitial damage (TID) in glomerulonephritis (GN) remains uncertain. Hence, the objective of this study is to clarify whether TID is associated with glomerular damage, and whether the damage at the tubulointerstitial compartment is more severe in hypertensive patients. The study included retrospectively consecutive patients referred to the Nephrology Unit with diagnoses of primary glomerulonephritis, lupus nephritis (LN), and nephroangiosclerosis (NAS) at biopsy. At least six glomeruli per biopsy were analysed through light and immunofluorescence microscopy. Global glomerulosclerosis (GGS%), TID, and arteriolar hyalinosis (AH) were used as markers of CKD severity. Of the 448 patients of the cohort, 403 received a diagnosis of GN, with the remaining being diagnosed with NAS. Hypertension was found in 52% of the overall patients, with no significant differences among those with GN, and reaching 88.9% prevalence rate in NAS. The hypertensive patients with GN had more marked damage in glomerular and tubular compartments than normotensives independently of the amount of proteinuria. Moreover, hypertension and GGS% were found to be strongly associated with TID in GN. In GN patients, not only the severity of glomerular damage but also the extent of TID was associated with high blood pressure.

## 1. Introduction

In 1968, Risdon et al. first reported a significant relationhip between the extent of tubular damage and creatinine clearance [1]. After this first report, the evaluation of tubulointerstitial damage (TID) in kidney biopsies progressively gained attention until the recognition of TID as the major histological indicator of progression in several chronic kidney diseases (CKD), irrespective of the initial injury that caused kidney damage. However, despite such widespread belief, most evidences came from experimental studies or, when in humans, from small size studies with patients suffering with specific forms of kidney diseases, often with no adjustment for confounding variables [2,3,4,5,6,7]. Recently, the large Boston Kidney Biopsy Cohort Study, by examining more than 500 biopsies of native kidneys in patients with a large variety of diseases, showed that the severity of interstitial fibrosis and tubular atrophy were independent predictors of kidney failure even after adjustment for estimated glomerular filtration rate (eGFR) and proteinuria [2], supporting the TID as the major determinant for CKD progression.

The key role of arterial hypertension in CKD progression is widely recognized. The hypertensive kidney disease is known as nephroangiosclerosis characterized by arteriolar hyalinosis and glomerulosclerosis [8], but the involvement of the tubulointerstitial compartment found in some experimental models suggested that TID could be associated with the glomerular damage, thereby, contributing to the kidney damage of hypertension [9]. However, the contribution of arterial hypertension to TID in glomerulonephritis (GN) remains uncertain. Hence, taking advantage of a large dataset of kidney biopsies from patients presenting with proteinuria, we investigated whether TID was associated with the glomerular damage and/or also with high blood pressure in glomerulonephritis.

## 2. Materials and Methods

### 2.1. Patients

All patients attending the Nephrology and Dialysis Unit of San Carlo Borromeo Hospital, Milan, Italy, between January 1992 and April 2006 who had renal biopsy diagnoses of primary glomerulonephritis, lupus nephritis (LN), and nephroangiosclerosis (NAS) were consecutively included in the study. Inclusion criteria were (a) persistent non-nephrotic proteinuria (PP), defined as proteinuria <3.5 g/24 h and normal serum albumin, or nephrotic syndrome (NS) (proteinuria ≥3.5 g/24 h and/or serum albumin <3.0 g/dL); (b) absence of previous nephrotic syndrome in the patients with PP; (c) typical features of glomerulonephritis or nephroangiosclerosis at light and immunofluorescence microscopy; (d) no clinical, imaging, or laboratory signs of secondary GN except for LN; (e) at least six glomeruli in the biopsy. High blood pressure (BP) was defined as office BP ≥140/90 mmHg at least on three occasions or antihypertensive treatment [10]. CKD was graded following classification of Kidney Disease Improving Global Outcomes (KDIGO) [11].

At follow-up, the outcomes were progression to end-stage renal disease (ESRD) or remission. In NS patients, remission was established as complete if proteinuria was ≤0.30 g/24 h, or partial if proteinuria was ≤2.0 g/24 h; in PP patients, remission was assessed if both proteinuria ≤0.30 g/24 h and normal renal function were found at the last observation.

The study complied with the Declaration of Helsinki and the local Institutional Reviw Board (IRB). All patients gave their written informed consent to the renal biopsy and use of data for scientific purposes.

### 2.2. Laboratory and Histology

Proteinuria was measured in 24 h urine collection and second morning urine sample by the Coomassie blue method (modified with sodium-dodecyl-sulphate) and expressed as 24 h proteinuria and protein creatinine ratio (mg urinary protein/g urinary creatinine). Serum and urinary creatinine were measured enzymatically and expressed in mg/dL. Serum and urinary IgG, transferrin, α2-macroglobulin (α2m), albumin, and α1-microglobulin (α1m) were measured by immunonephelometry and expressed as urinary protein/creatinine ratios. NAG (N-acetyl-β-glucosaminidase) was measured by a colorimetric assay performed using 3-cresolsulfonphthaleyn-N-acetyl-β-D-glucosaminide that is hydrolized by NAG with the release of 3-cresolsulfonphthaleyn sodium salt, which is measured photometrically at 580 nm on a HITACHI Instrument; the results were expressed in units/g urinary creatinine. Estimated glomerular filtration rate (eGFR) was measured by the Chronic Kidney Disease Epidemiology Collaboration (CKD-EPI) formula [12]. The following markers of CKD severity were considered: (1) percentage of glomeruli with global glomerulosclerosis (GGS%); (2) extent of tubulointerstitial damage (TID) evaluated semi-quantitatively by a score ranging from 0 to 6, with tubular atrophy, interstitial fibrosis, and inflammatory cell infiltration each graded as 0, 1, or 2 if absent, focal, or diffuse; (3) extent of arteriolar hyalinosis (AH) evaluated semi-quantitatively by a score: 0, 1, 2, 3 if absent, focal, diffuse, diffuse with lumen reduction, respectively.

### 2.3. Statistics

Normal distribution was assessed with the Kolmogorov–Smirnov test, and quantitative variables that showed a skewed distribution underwent appropriate transformation to achieve normal distribution. One-way ANOVA followed by Bonferroni’s post hoc test, or *t* test, and Mann–Whitney or Kruskall–Wallis were used to compare quantitative variables among/between groups for variables with or without normal distribution, respectively. Distribution of categorical variables was compared by chi-square analysis.

Ordinal regression analysis to identify factors associated with TID used the test of parallel lines to assess whether the assumption of proportional odds was satisfied, Pearson P to evaluate the goodness of fit test, and Nagelkerke value to assess the proportion of variation of the outcome (TID) explained by factors and covariates. For regression analysis, TID score was cropped into three levels corresponding to absence of TID, focal TID, and diffuse TID. Significance was set at *p* < 0.05.

## 3. Results

**Figure 1:**
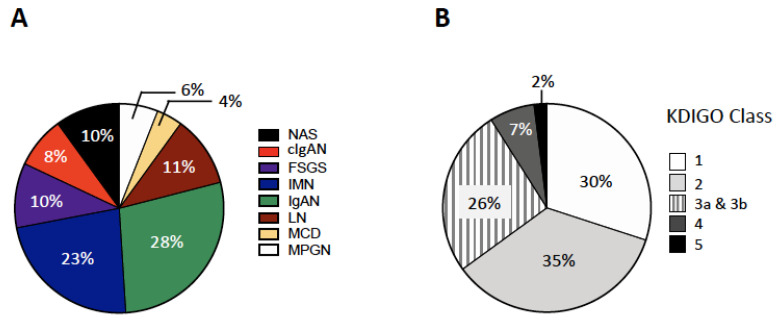
Distribution of nephropathies at histological analysis in the overall cohort (Panel **A**) and distribution of patients by Kidney Disease Improving Global outcomes (KDIGO) classification in the overall cohort (Panel **B**). IgAN: IgA nephropathy; IMN: idiopathic membranous nephropathy; LN: lupus nephritis; FSGS: focal segmental glomerulosclerosis; cIgAN:screscentic IgA nephropathy; MPGN: membranoproliferative glomerulonephritis; MCD: minimal change disease; NAS: nephroangiosclerosis.

Demographic characteristics of the entire cohort of patients (*n* = 448) at baseline are shown in Table 1. At biopsy, the diagnoses were IgA nephropathy (IgAN, *n* = 127, 28%), idiopathic membranous nephropathy (IMN, *n* = 100, 23%), lupus nephritis (LN, *n* = 49, 11%), focal segmental glomerulosclerosis (FSGS, *n* = 46, 10%), crescensic IgA nephropathy (cIgAN, *n* = 37, 8%), membranoproliferative glomerulonephritis (MPGN, *n* = 26, 6%), and minimal change disease (MCD, *n* = 18, 4%). In 45 patients (10%), the diagnosis was nephroangiosclerosis (NAS) (*n* = 45, 10%) (Table 1; Figure 1A).

Males were prevalent in the entire cohort (M/F, 58%/42%) and in each GN group except for LN, which was characterized by a female prevalence (*p* < 0.001 vs. any other group). The overall mean age was 43 years; NAS patients were older than those of any other GN group (Table 1). Hypertension was found in 52% of the overall patients, with no significant differences in prevalence rates between GN groups. The majority of NAS patients were hypertensives (Table 1). Distribution of patients by KDIGO classification showed prevalence of classes 1–3 (Figure 1B), with a mean eGFR of 72 mL/min/1.73 m^2^. Nephrotic syndrome was found in 46% in the entire cohort (Table 1), with prevalence rates ranging from 1.6% in IgAN to 100% in MCD (Table 1).

### 3.1. Kidney Damage in Hypertensive and Normotensives Patients with GN

Hypertensive patients with glomerulonephritis had lower eGFR than normotensive patients (*p* < 0.001) and more prominent damage in glomerular and tubulointerstitial compartments, in addition to higher excretion of albumin/creatinine, IgG/creatinine, and α1-microglobulin/creatinine (*p* < 0.001 for all) (Table 2).

Patients with hypertension were older (*p* < 0.01) than normotensive patients. When considering GN patients for nephrotic (NS) or persistent proteinuria (PP), no significant difference was found in eGFR.

In GN patients with nephrotic syndrome, the co-existence of hypertension was associated with lower eGFR (*p* < 0.001 vs. normotensives) and higher excretion of IgG and α1-microglobulin (*p* < 0.001 vs. normotensives) (Table A1). GN patients with persistent proteinuria (PP), if hypertensives, had lower eGFR (*p* < 0.001) and higher protein excretion than normotensives (*p* = 0.007) (Table A1). eGFR was found to be lower in hypertensives than in normotensives also when considering each GN group (Table A2).

### 3.2. Kidney Damage in Hypertensive and Normotensives Patients with NAS

No significant difference was observed between hypertensive and normotensive patients in the NAS group (Table 2); NAS patients with nephrotic proteinuria were older (*p* < 0.02) and had lower eGFR (*p* < 0.05).

### 3.3. GGS and TID

**Figure 2:**
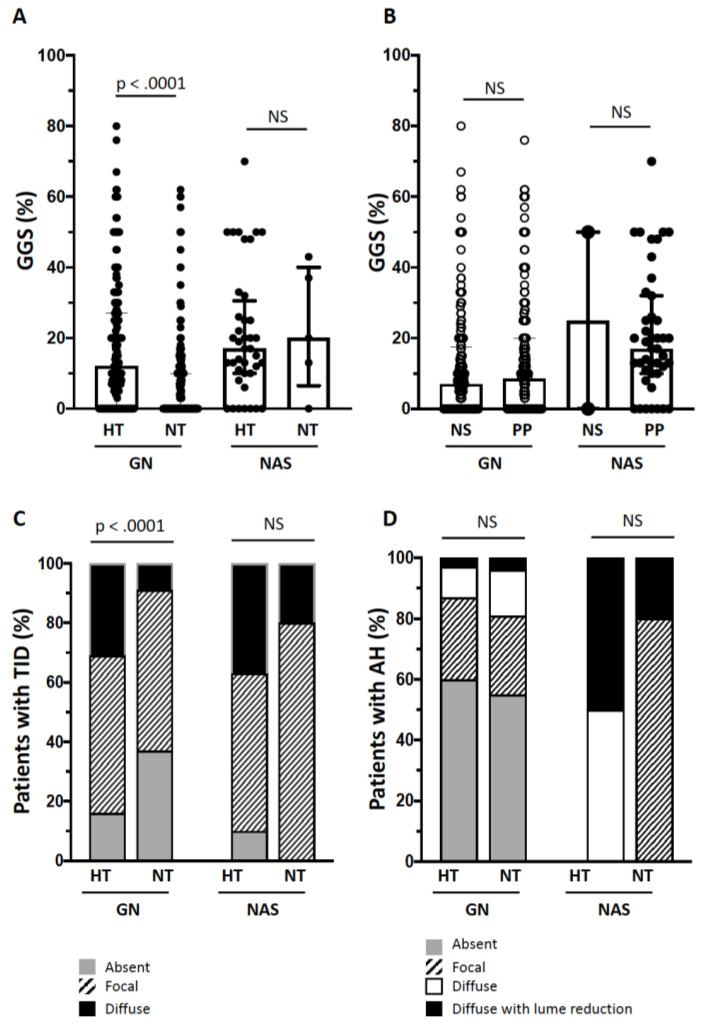
Global glomerulosclerosis (GGS%) in the patients with hypertension (HT) or normal blood pressure (NT) in the subgroups of glomerulonephitis (GN) and nephroangiosclerosis (NAS) (Panel **A**). GGS% in the patients with nephrotic syndrome (NS) or persistent proteinuria (PP) in the subgroups of GN and NAS (Panel **B**). Prevalence of tubulointerstitial damage (TID) or arteriolar hyalinosis (AH) scores in the HT or NT patients in the subgroups of GN and NAS (Panels **C** and **D**, respectively).

In GN patients, glomerular damage expressed as GGS% was more pronounced when hypertension coexisted (*p* < 0.0001) (Figure 2A), but no difference was found between NS and PP (Figure 2B). A larger proportion of GN hypertensive patients showed more severe TID than normotensives (*p* < 0.0001) (Figure 2C), whereas hypertensives and normotensives did not differ for AH score (Figure 2D).

**Figure 3:**
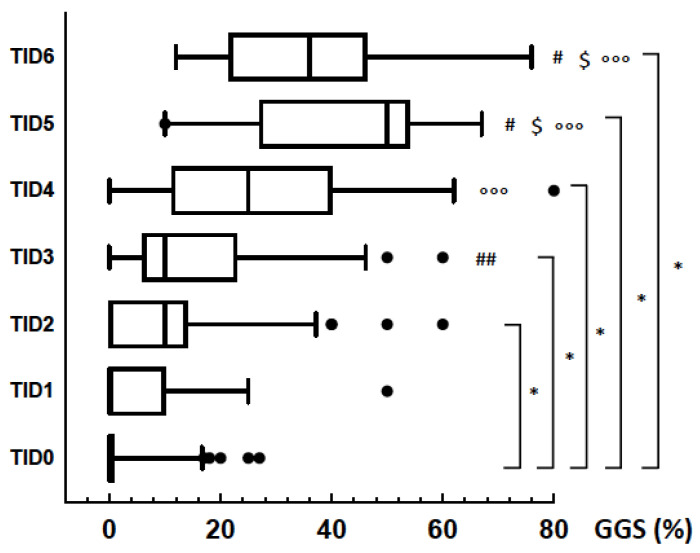
Distribution of TID score by GGS% in the patients with glomerulonephitis. *: *p* < 0.001 vs. TID 0; #: *p* < 0.0001 vs. TID 1; ##: *p* = 0.002 vs. TID 1; °°°: *p* < 0.001 vs. TID 2; $ *p* = 0.04 vs. TID 3.

**Figure A1:**
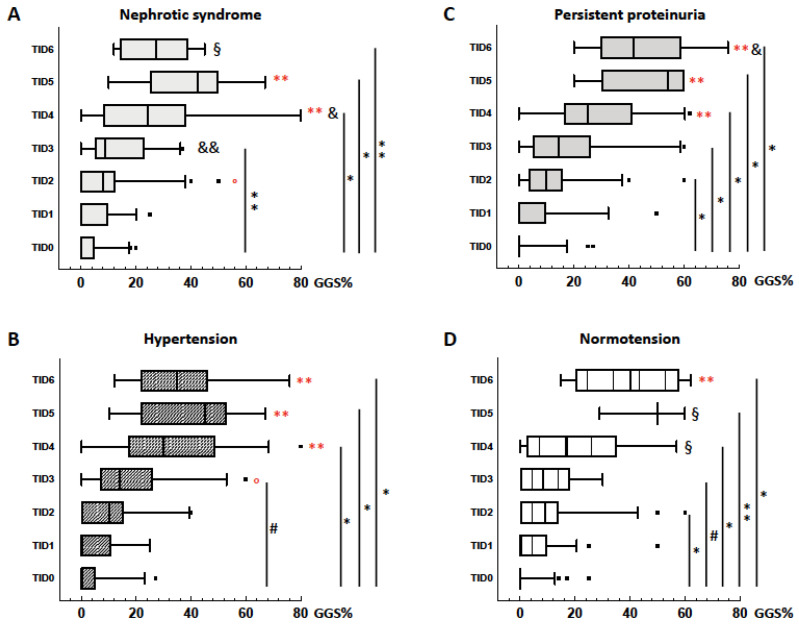
Severity of tubulointerstitial damage (TID) by global glomerulosclerosis (GGS%) in the patients with nephrotic syndrome (Panel **A**), arterial hypertension (Panel **B**), persistent proteinuria (Panel **C**), and normotension (Panel **D**). *: *p* < 0.0001 vs. TID 0; #: *p* = 0.03 vs. TID 0; **: *p* = 0.01 vs. TID 0; §: *p* = 0.04 vs. TID 1; **: *p* = 0.01 vs. TID 1; & *p* = 0.02 vs. TID 2; °: *p* = 0.02 vs. TID 5; &&: *p* = 0.04 vs. TID 5.

TID score increased along with GGS% in GN patients (Figure 3), and this trend was seen in patients with or without hypertension, with or without nephrotic syndrome (Figure A1).

In NAS patients, GGS% and TID did not differ between hypertensive and normotensive patients, or NS and PP (Figure 2) but, because there were only five normotensives, such a result cannot be conclusive.

At ordinal regression analysis, we found that hypertension and GGS% were the factors associated with TID in GN.

Model 5 was not considered because the test of parallel lines showed that assumption of proportional odds was not satisfied.

By inserting age, sex, hypertension, and presence of nephrotic syndrome in the model, again a strong association between hypertension and TID was found (model 1). The odds of developing TID in patients with GN and hypertension was 3.61 (95% CI, 2.35–5.55) times that in normotensives (Wald X^2^ = 34.43, *p* < 0.0001), with hypertension explaining 11% of the variation between TID scores (Nagelkerke value = 0.115) (Table 3). Leaving only hypertension in the model 2, we found similar results (OR = 3.61, 95% CI, 2.35–5.55), supporting that hypertension is associated with TID. By adding GGS% to hypertension in model 3, we found that GGS% explains 44% of the variation between TID scores. A similar result was obtained from model 4, which considered GGS% alone (Table 3).

When considering GN patients by coexistence of arterial hypertension or not, we found that TID was strongly associated with GGS% and AH score in both normotensives (models 1a and 2a) and hypertensives (models 1b and 2b) (*p* < 0.0001 both) (Table 3).

In patients with NAS, the odds of developing TID was 1.04 (95% CI, 1.01–1.08) times in those showing GGS% than in those having no GGS (Wald X^2^ = 4.97, *p* < 0.03) (Table 3).

When we performed the regression analysis by omitting the advanced CKD stages, i.e., stages 4 and 5 (corresponding to 9% of GN patients, in whom a pre-existing unknown tubulointerstitial damage could occur), we found very similar results, supporting the association of GGS and high blood pressure with TID in GN patients.

### 3.4. Follow-Up

We considered 359 patients (NS, *n* = 177; PP, *n* = 182) who had a follow-up longer than 2 months (NS: 63 (29–121), PP: 60 (34–88), median (IQR) months). The hypertensive patients developed ESRD more frequently than normotensives (*p* = 0.0037). When considering the patients by immunosuppressive treatment, no difference was found between hypertensives and normotensives treated with steroids and cyclophosphamide (*p* = 0.07). However, when analyzing the patients not undergoing immunosuppressive treatment, a greater proportion of hypertensives developed ESRD than did normotensives (*p* = 0.02).

## 4. Discussion

CKD is a global health challenge with an impact on morbidity and quality of life [13,14] leading to more than one million deaths per year [15,16,17]. Since a deeper knowledge of the mechanisms underlying CKD could allow identification of biomarkers and tailored treatments, we investigated the impact of arterial hypertension on kidney function and the involvement of the tubulointerstitial compartment in GN. By exploring a large database of patients undergoing renal biopsy because of proteinuria, we found that the hypertensive patients with glomerulonephritis had more prominent damage in both glomerular and tubular compartments than normotensive patients independently of the amount of proteinuria.

As similarly reported in large biopsy databases of CKD patients, we found that IgAN was the most common primary GN, and that IgAN and LN were prevalent in males and females, respectively [18,19,20,21]. Consistently with previous surveys [22,23,24,25,26], hypertension was a frequent comorbidity in the CKD patients of the present study.

eGFR was lower in the hypertensive than in normotensive patients, and this was found in each GN subtype, supporting the role of hypertension in deteriorating the kidney function in GN. In contrast, no significant difference in eGFR was seen between nephrotic syndrome and persistent proteinuria. However, in patients with PP, hypertension was associated with lower eGFR and higher excretion of proteins, and, in those with nephrotic syndrome, hypertension was associated with lower eGFR and higher excretion of total proteins, IgG, and α1-microglobulin, further supporting the role of hypertension in worsening kidney damage in GN.

A recent report of the Atherosclerosis Risk in Communities (ARIC) study has shown that, in a general population cohort, hypertension status is associated with faster kidney function decline and that the slope of eGFR decline can be attenuated by antihypertensive treatment [24]. Our study, specifically focused on GN, consistently documents that coexistence of hypertension in patients with GN implies heavier kidney damage and more frequent development of ESRD when compared to normotensives.

Another finding of the present study was that tubular damage is greater in GN if hypertension coexists. By occupying 80% of total kidney volume, the tubules represent the dominant compartment and, being tightly connected to the interstitium and vessels that interact with the mesangium, they are easily vulnerable to the events that injure glomeruli [27]. Hence, a structural derangement may occur in the tubulointerstitial compartment regardless of the site of first injury, thereby explaining why interstitial fibrosis and tubular atrophy were found to be independent predictors of kidney failure in a large kidney biopsy study [2].

Only in last decade, experimental [9] and clinical studies [28] have documented that hypertension can cause TID in addition to glomerular and perivascular damage. In the SPRINT Study, BP lowering resulted not only in slower eGFR decline but also lower levels of β2-microglobulin and α1m [28]. Moreover, the levels of markers of tubular damage not only predicted Acute Kidney Injury [29] but also cardiovascular events and mortality [30].

Whether high blood pressure contributes to the development of TID in GN remained to be established. In 2006, Ikee et al., examining biopsies from patients with IgA GN, not only observed that TID correlated with GGS% but also that it was more pronounced in the hypertensive patients, suggesting, but not proving, that hypertension worsened the damage at both tubular and glomerular levels in IgA GN [31]. Later, in another cross-sectional study, Haruhara et al. found that histopathological findings assessed with the Oxford classification well associated with circadian blood pressure changes in IgA GN, but, again no causal relationship could be proved [32]. Whether hypertension is the driver in worsening TID, or is a marker of severe GN, remains uncertain also in the present study.

### Strengths and Limitations

Strengths of the study are (1) the large cohort of GN patients included all frequent GN subtypes; (2) the biopsy analysis was performed by one pathologist who used an identical procedure and, moreover, included reproducible and quantitative measurements of GGS% and semi-quantitative measurement of TID and AH scores.

The limitations of the study are as follows: (1) Since arterial hypertension can be either the cause or consequence of CKD and both hypertension and CKD can be silent for years [26,33], making difficult to establish their onset, it is difficult to understand the causal relationship between them [34]. The present study, being observational, leaves open the issue of whether hypertension is the cause or consequence of CKD induced by GN. (2) In our dataset, data on antihypertensive treatment were available only in part, thereby preventing any consideration of the role of drugs on TID. However, the lack of this type of information does not challenge our conclusion that hypertension has an impact on TID. In fact, since antihypertensive drugs as ACE inhibitors can slow progression of CKD and reduce proteinuria, they could have reduced, not magnified, the impact of hypertension on TID. (3) Information on obesity and diabetes mellitus, which are relevant factors in the development of kidney damage, was often missing in our database, preventing any consideration of this issue.

## 5. Conclusions

The present study, by examining a large cohort of GN patients, has provided data that collectively support a role of hypertension in the tubular decline in most common primary GN. The hypertensive patients more frequently developed focal or diffuse TID than normotensives did and had greater excretion of low molecular weight proteins, such as α1m. In the hypertensives, the tubular damage was greater than that in normotensives even if we separately consider the subgroups of patients with nephrotic syndrome and persistent proteinuria.

## Figures and Tables

**Table 1:** Demographic characteristics at baseline of patients with glomerulonephritis (GN) and nephroangiosclerosis (NAS).

Variable	All Patients	IgAN	IMN	LN	FSGS	cIgAN	MPGN	MCD	NAS
	(*n* = 448)	(*n* = 127)	(*n* = 100)	(*n* = 49)	(*n* = 46)	(*n* = 37)	(*n* = 26)	(*n* = 18)	(*n* = 45)
Sex	260 (58)	83 (65)	61 (61)	8 (16) ***	24 (52) *	24 (65)	14 (54)	11 (61)	35 (78)
M (*n*, %)									
Age	43 ± 17	42 ± 17 ***	48 ± 18 *	33 ± 14 ***	39 ± 17 ***	30 ± 11 ***	39 ± 15 ***	42 ± 20 **	57 ± 13
(years)									
eGFR	72 ± 30	71 ± 27	71 ± 30	78 ± 32 *	82 ± 30 *	62 ± 33	67 ± 38	90 ± 29 **	64 ± 22
(mL/min/1.73 m^2^)									
Hypertension	233 (52)	55 (43.3) ***	56 (56) ***	22 (44.9) ***	25 (54.3) ***	20 (54.1) ***	17 (65.4) *	6 (33.3) ***	40 (88.9)
(*n*, %)									
Nephrotic syndrome	206 (46)	2 (1.6)	80 (80.0) ***	29 (59.2) ***	40 (87) ***	15 (40.5) ***	20 (76.9) ***	18 (100) ***	2 (4.4)
(*n*, %)									

eGFR: estimated glomerular filtration rate; IgAN: IgA nephropathy; IMN: idiopathic membranous nephropathy; LN: lupus nephritis; FSGS: focal segmental glomerulosclerosis; cIgAN: cresensic IgA nephropathy; MPGN: membranoproliferative glomerulonephritis; MCD: minimal change disease. Mean ± SD. * *p* = 0.05, ** *p* < 0.01, *** *p* < 0.001, vs. NAS.

**Table 2:** Renal function in GN and NAS patients with/without arterial hypertension.

Variable	GN		*p* HT vs. NT	NAS		*p* HT vs. NT
	HT (*n* = 201)	NT (*n* = 202)		HT (*n* = 40)	NT (*n* = 5)	
Sex (M, %)	125 (62)	100 (50)	0.01	32 (80)	3 (60)	0.30
Age (years)	43 ± 18	38 ± 17	0.01	57 ± 2	54 ± 7	0.58
eGFR (mL/min/1.73 m^2^)	56 (35–78)	89 (72–102)	<0.001	62 ± 3	76 ± 22	0.20
U-proteins/creatinine (mg/µg)	2406	880	<0.001	464	809	0.77
	(750–4978)	(220–2550)		(515–1027)	(0–1759)	
U-IgG/creatinine (mg/µg)	99	37	<0.001	29	18	0.58
	(34–275)	(10–102)		(28–94)	(0–66)	
U-albumin/creatinine (mg/µg)	1948	626	<0.001	350	495	0.61
	(588–4214)	(130–2398)		(389–845)	(0–1654)	
U-α1-microglobulin/creatinine (mg/µg)	26	9	<0.001	9	15	0.57
	(11–55)	(4–21)		(11–25)	(4–22)	
U-transferrin/creatinine (mg/µg)	125	43	<0.001	19	21	0.94
	(30–325)	(10–197)		(21–47)	(0–77)	
U-NAG/creatinine (IU/g)	9.76	5.9	<0.001	5	7	0.45
	(4.69–20.96)	(2.93–11.41)		(5–8)	(3–13)	

Mean ± SD, or median (Interquartile range (IQR)), as appropriate. GN: glomerulonephritis; NAS: nephroangiosclerosis.

**Table 3:** Ordinal regression analysis in GN.

Model	Disease	Factors and Covariates	Test Parallel Lines	Goodness of Fit Test (Pearson P Deviance P)	Pseudo *R*-Square (Nagelkerke)	Associated Variable(s)	OR (95% CI)	Wald	*p*
1	GN	Age	0.02	0.610 0.362	0.11	HT	3.61 (2.35–5.55)	34.43	<0.001
		Sex							
		HT							
		Nephrotic syndrome							
2	GN	HT	0.327	0.329	0.10	HT	3.48 (2.31–5.25)	35.21	<0.001
				0.327					
3	GN	GGS	0.240	0.981	0.44	GGS	1.11 (1.08–1.13)	94.32	<0.001
		HT		0.981		HT	1.91 (1.22–3.00)	7.99	
4	GN	GGS	0.088	0.867	0.42	GGS	1.11 (1.09–1.13)	106.82	<0.001
				0.869					
5	GN	GGS	0.033	0.738 0.995	0.50	GGS	1.09 (1.07–1.11)	63.61	<0.001
		HT				HT	1.64 (1.04–2.60)	4.51	0.034
		AH				AH	0.055 (0.01–0.3)	13.99	<0.001
1a	GN NT	GGS	0.100	0.502	0.31	GGS	1.11 (1.08–1.50)	40.50	<0.001
				0.638					
2a	GN NT	AH	0.402	0.924	0.26	AH	5.35 (3.16–9.04)	39.12	<0.001
				0.883					
1b	GN HT	GGS	0.496	0.996	0.43	GGS	1.10 (1.07–1.13)	53.73	<0.001
				0.991					
2b	GN HT	AH	0.744	0.0.92	0.25	AH	3.46 (2.35–5.11)	39.14	<0.001
				0.096					
1c	NAS	GGS	0.058	0.851	0.13	GGS	1.04 (1.01–1.08)	4.97	0.026
				0.726					

AH: arteriolar hyalinosis; GGS: global glomerulosclerosis. Dependent variable: TID. Assumption of proportional odds is satisfied if test of parallel lines >0.05; goodness-of-fit test: the model fits well if *p* > 0.05.

## Appendix A

**Table A1:** Renal function in GN patients with/without arterial hypertension.

Variable	GN and NS		*p* HT vs. NT	GN and PP		*p* HT vs. NT
	HT (*n* = 122)	NT (*n* = 82)		HT (*n* = 79)	NT (*n* = 120)	
Sex (M, %)	69 (57)	36 (44)	0.08	56 (71)	64 (53)	0.01
Age (years)	43 ± 18	38 ± 16	0.01	43 ± 16	39 ± 16	0.11
eGFR (mL/min/1.73 m^2^)	57 ± 30	91 ± 26	0.01	60 ± 24	85 ± 25	0.01
U-proteins/creatinine (mg/µg)	4201	3277	0.31	585	332	0.01
	(2760–6224)	(1835–5913)		(214–1176)	(114–848)	
U-IgG/creatinine (mg/µg)	193	102	0.01	31	13	0.01
	(91–422)	(52–193)		(10–60)	(6–38)	
U-albumin/creatinine (mg/µg)	3516	2978	0.11	403	224	0.02
	(2157–5155)	(1531–5107)		(108–930)	(54–562)	
U-α1-microglobulin/creatinine (mg/µg)	43	21	0.01	10	5	0.01
	(24–87)	(10–39)		(4–19)	(3–11)	
U-transferrin/creatinine (mg/µg)	262	232	0.34	22	10	0.02
	(148–393)	(116–424)		(6–51)	(3–39)	
U-NAG/creatinine (IU/g)	16.89	13.00	0.34	4.61	3.5	0.01
	(9.3–27.5)	(6.75–21.31)		(3.08–7.88)	(2.2–6.3)	

Mean ± SD, or median (95% CI) as appropriate. HT: hypertension; NT: normal blood pressure.

**Table A2:** Renal function in the patients with GN and with/without hypertension.

Variable		cIgAN	MPGN	IMN	IgAN	LN	FSGS	MCD
		(*n* = 37)	(*n* = 26)	(*n* = 100)	(*n* = 127)	(*n* = 49)	(*n* = 46)	(*n* = 18)
HT/NT (*n*)	HT	14/6	11/6	38/18	40/15	2/20	16/9	4/2
	NT	10/7	3/6	23/21	43/29	6/21	8/13	7/5
Age (years)	HT	32 ± 12	40 ± 15	49 ± 18	43 ± 17	36 ± 16	48 ± 18 ***	50 ± 20
	NT	28 ± 9	37 ± 15	47± 18	42 ± 17	31 ± 10	29 ± 7 **	38 ± 19
eGFR (mL/min/1.73 m^2^)	HT	40.50 ***	46.00 **	58.50 ***	59.00 ***	70.50 *	61.00 ***	65.00
		(17.50–52.25)	(18.50–87.50)	(32.00–78.00)	(45.00–70.00)	(25.25–92.50)	(43.50–94.00)	(48.25–90.25) *
		94.00	96.00	90.50	79.00	91.00	102.00	98.00
		(67.50–100.00)	(84.50–117.50)	(75.25–103.50)	(61.00–93.75)	(76.00–102.00)	(88.50–113.50)	(88.00–128.50)
U-proteins/creatinine (mg/µg)	HT	1804 **	4859	3801 **	407	2820 ***	5359	4277
		(965–3829)	(1341–8490)	(2188–6571)	(131–850) *	(1483–4667)	(2796–9052)	(3270–3135)
	NT	545	1761	2438	204	907	3000	4664
		(228–1395)	(611–5978)	(1297–4287)	(99–532)	(524–1781)	(650–7697)	(2401–6643)
U-IgG/creatinine (mg/µg)	HT	113 *	199 *	181 ***	21 **	125 *	195 *	116
		(48–289)	(69–858)	(67–403)	(7–59)	(71–386)	(79–383)	(76–270)
	NT	32	45	68	10	68	102	94
		(12–76)	(18–197)	(28–131)	(5–28)	(15–149)	(29–186)	(52–148)
U-albumin/creatinine (mg/g)	HT	1783 ***	3973	3355 **	313 *	2191 ***	4656	4046
		(814–3010)	(1234–5855)	(1788–5103)	(47–704)	(988–3941)	(2070–6556)	(2804–5186)
	NT	432	1735	2280	126	624	2621	4398
		(141–1292)	(497–4461)	(1143–3664)	(32–468)	(266–1259)	(474–6414)	(1941–6225)
U-α1-microglobulin/creatinine (mg/g)	HT	32 ***	78 **	42 ***	10 **	26	41 *	30
		(16–49)	(16–104)	(25–87)	(4–18)	(13–57) *	(18–62)	(19–42)
	NT	6	5	15	5	11	21	17
		(2–12)	(3–42)	(7–33)	(2–11)	(5–25)	(7–40)	(15–31)
U-transferrin/creatinine (mg/g)	HT	108 **	246	264	13 *	151 ***	335	319
		(38–189)	(55–507)	(117–371)	(3–32)	(92–231)	(155–572)	(234–425)
	NT	19	121	189	6	43	211	332
		(8–65)	(25–437)	(66–317)	(2–23)	(19–87)	(36–536)	(208–464)
U-NAG/creatinine (IU/g)	HT	10.32 **	18.75	15.81 **	4.05 *	12.35	14.89	7.61
		(6.62–16.63)	(4.67–36.21)	(9.90–26.14)	(2.82–6.36)	(6.47–31.02)	(9.02–26.72)	(5.61–15.51)
	NT	4.68	5.55	9.86	2.94	7.66	10.00	12.01
		(3.19–6.53)	(2.66–38.41)	(5.44–18.01)	(2.08–5.33)	(5.77–13.36)	(5.58–25.81)	(7.04–17.3)

* *p* < 0.01 vs. NT; ** *p* < 0.001 vs. NT; *** *p* < 0.0001 vs. NT. HT: hypertension; NT: normotension.
